# Acute FeNO and Blood Pressure Responses to Air Pollution Exposure in Young Adults during Physical Activity

**DOI:** 10.3390/ijerph17239012

**Published:** 2020-12-03

**Authors:** Krzysztof Kocot, Kamil Barański, Edyta Melaniuk-Wolny, Elwira Zajusz-Zubek, Małgorzata Kowalska

**Affiliations:** 1 Department of Epidemiology, Faculty of Medical Sciences in Katowice, Medical University of Silesia in Katowice, 40-752 Katowice, Poland; kbaranski@sum.edu.pl (K.B.); mkowalska@sum.edu.pl (M.K.); 2 Department of Air Protection, Faculty of Energy and Environmental Engineering, Silesian University of Technology in Gliwice, 44-100 Gliwice, Poland; edyta.melaniuk-wolny@polsl.pl (E.M.-W.); elwira.zajusz-zubek@polsl.pl (E.Z.-Z.)

**Keywords:** exercise, air pollution, FeNO, blood pressure, young adults

## Abstract

During physical exercise, the absorbed dose of air pollutants increases. Acute effects of exposure to air pollutants during exercise in healthy young adults remain poorly documented. The aim of this study was to assess the acute responses in fractionated exhaled nitric oxide (FeNO) and blood pressure to air pollution exposure during exercise in young adults with different physical activity levels (low or high). In this study, 76 healthy university students participating in physical activity classes (low level of physical activity) and attending sports training (high level of physical activity) completed two indoor exercise trials when air pollutant concentrations were high (exposure trial) and when the quality of the air was good (control trial). We monitored indoor particulate matter with diameter <10 µm and <2.5 µm (PM_10_ and PM_2.5_) and outdoor PM_10_, nitric oxides (NO_2_, NO_x_, NO), and sulfur dioxide (SO_2_) concentrations. Systolic and diastolic blood pressure (SBP and DBP), heart rate (HR), oxygen saturation (SpO_2_)_,_ and FeNO were measured at baseline and after 45–60 min of physical activity. There were no significant differences between physiological responses to training performed under different exposure conditions in blood pressure, HR, and SpO_2_. Significant positive correlations between post-exercise ΔFeNO during exposure trials and ambient air pollutants were found. FeNO increase during the exposure trial was associated with a higher physical activity level and higher outdoor PM_10_ and NO_2_ concentrations. In young and healthy adults, some differences in physiological responses to physical activity between polluted and control environments could be observed. Participants with a high physical activity level were more likely to have an increase in FeNO after exercise in a polluted environment but not after the control exercise trials.

## 1. Introduction

Physical activity has a well-established role in the prevention of cardiovascular diseases [1,2], as well as many other chronic conditions including diabetes [3] and musculoskeletal diseases [4]. On the other hand, exercising in a polluted urban environment can result in an increased absorbed dose of air pollutants [5,6]. Particularly in susceptible groups, short term exposure to air pollution increases the risk of acute cardiovascular events and exacerbations of respiratory diseases [7,8,9]. Studies showed, for example, that walking near a busy road was related to a deterioration in cardiovascular and respiratory function in patients with chronic obstructive pulmonary disease (COPD) and healthy older adults [10].

However, the negative impact of air pollutant exposure during exercise on the cardiovascular and respiratory system could be observed even in healthy adults [11,12]. Exercising in a polluted environment resulted in, among others, increased blood pressure [13], decreased airflow [14,15], and increased pulmonary inflammation [16]. Inflammation plays important role in the pathophysiology of the negative health impact of air pollution [8]. The release of inflammatory, pro-oxidative and hemodynamically active mediators leads to autonomic imbalance and vasoconstriction, which results in elevated blood pressure [17], among other effects. Both long and short-term exposure to particulate matter air pollution is associated with an increase in systolic and diastolic blood pressure (SBP and DBP) [18].

An important biomarker of pulmonary inflammation is fractionated exhaled nitric oxide (FeNO). Increased FeNO values indicate eosinophilic and allergic airway inflammation, and therefore its measurement is used in the diagnosis and management of asthma [19]. FeNO level is also affected by air pollution, even in non-asthmatics [20]. Research from China showed a significant decrease in FeNO and several other biomarkers of pulmonary and systemic inflammation, systolic blood pressure and heart rate in healthy adults in response to a decrease in air pollution during the 2008 Beijing Olympics [21]. In contrast to air pollution, regular physical activity may lessen airway inflammation and decrease FeNO levels [22]. Consistent with this fact, it has been noted that in healthy people, exercise may lessen the acute negative impact of air pollution [16,23]. In contrast, other studies showed no negative respiratory effects of short term exposure during exercise in healthy adults [24,25]. Health assessment studies indicated that in normal conditions, the positive impact of exercise outweighs the risk related to air pollution exposure [26]. Nevertheless, the relationship between air pollution and exercise is complex and not fully understood or documented. Different exposure levels in available studies may be partly responsible for some contrasting results [11,27]. Most of the studies addressing the acute effects of exposure to air pollution during exercise come from Western Europe and North America [10,13,14,15,16,24,25]. Poland is characterized by significantly higher levels of air pollutants, especially during the winter season [28]. This is the reason why we decided to perform the study, in which the main aim was to examine the acute cardiovascular and inflammatory responses of young and healthy adults to air pollution exposure during physical training.

## 2. Materials and Methods

### 2.1. Study Site

The study took place between November 2019 and March 2020 in Gliwice, Poland. The city of Gliwice (180,000 inhabitants) is a part of the Upper Silesian Agglomeration, an area characterized by a high population density (366 persons/km^2^) and substantial air pollution problems, especially during the winter season. According to data from an official monitoring station located in Gliwice, the average annual level of particulate matter with diameter <10 µm (PM_10_) in 2018 was 40 µg/m^3^ and the Polish daily norm of 50 µg/m^3^ was exceeded during 92 days [29]. Upper Silesia is located in the south part of Poland, and lies in a physic-geographical region of Silesia-Kraków Upland with a temperate climate. The mean annual air temperature in 2019 was 10.2 °C and the annual precipitation ranged between 600 and 700 mm [30,31].

All training and health measurements were conducted in three sports halls of the Silesian University of Technology, located close to the university campus and the city center—New Hall (address: Kaszubska str. 28, Gliwice), OSIR Hall (Akademicka str. 26, Gliwice), and Konarskiego Hall (Konarskiego str. 22, Gliwice). During the study period, the ambient air temperature at the university campus ranged between 0.8–15.6 °C, and the relative air humidity varied from 52.9% to 93.6%.

### 2.2. Participants

The participants were healthy volunteers recruited from students of the Silesian University of Technology who were attending obligatory physical education classes (low level of physical activity group) and members of the University Sports Association (Akademicki Związek Sportowy (AZS)) who were regularly participating in sports training (high activity level group). The inclusion criteria were informed consent, age of 18–30 years, absence of chronic cardiovascular or respiratory disease, and no current infection. Allergy, unless accompanied by asthma, was not considered as an exclusion criterion. Each volunteer answered questions on place of residence, housing conditions, current health status, and exercise patterns by completing the author’s questionnaire. Exercise patterns described in the questionnaire supported the choice of the low and high level of the physical activity group. Overall, 141 students were invited to the study; 90 agreed to participate (participation rate: 64%), and 84 completed two training trials with complete data available for 76 (due to measurement device flaws).

### 2.3. Study Design

The study had a case-crossover design. Each participant completed two training trials in different exposure conditions. The exposure trials took place during days with significantly increased levels of ambient air pollutants, while control trials were conducted when the air quality was good (defined as outdoor PM_10_ < 50 µg/m^3^, based on WHO Guidelines and the EU Air Quality Directive of the daily reference value). We planned the dates of trials according to the air pollution forecast provided by the Chief Inspectorate of Environmental Protection in Katowice. In case the two trials did not meet the criteria for exposure and control trials, a third session was realized. The two sessions with the largest difference in air pollution were then further analyzed. The trials were completed within the normal training schedule for each participant during physical education classes or training with the University Sports Association. The type of exercise was not influenced by the researchers and consisted mainly of volleyball and basketball in physical education classes and volleyball, basketball, and judo in University Sports Association training. This fact may be conceived as the primary limitation of the methods, as we cannot exclude that the exercise level differed between specific exercise sessions.

During each training, two measurements were conducted: at baseline and after the exercise session. Firstly, participants completed a short qualification questionnaire, then they were measured and weighed. Next, each participant had measurements taken of blood pressure, pulse oximetry, spirometry, and fractionated exhaled nitric oxide (FeNO). For blood pressure measurement, we used an Omron M2 Basic device (Omron Healthcare, Hoofddorp, Netherlands, declared measurement accuracy: ± 3 mmHg) and for pulse oximetry, we used a Pulsox-2 Oximeter (Konica Minolta, Tokyo, Japan, accuracy: ± 2% SpO_2_, ± 2 bpm). Participants performed spirometry in a sitting position, according to the European Respiratory Society (ERS) and American Thoracic Society (ATS) guidelines with an Easy One Air spirometer (NDD, Zurich, Switzerland, flow accuracy ± 2% or 0.020 L/s, volume accuracy ± 2% or 0.050 L). Each time, the participant repeated the maneuvers until they met the ERS/ATS acceptability and repeatability criteria or the maximal number of eight maneuvers was reached. For FeNO measurement, we used a Vivatmo Pro (Bosch, Gerlingen, Germany, accuracy ± 5 ppb for < 50 ppb, ± 10% for > 50 ppb). After completion of baseline measurements, participants attended their normal training. After 45 to 60 min, they were asked to return to the health measurement room and the same measurements were repeated.

All training took place indoors. During exercise, the indoor PM_2.5_ and PM_10_ levels were monitored in the sports hall with the use of a TSI SidePak AM520 (TSI, Shoreview, MN, USA, zero stability: ± 0.001 mg/m^3^ over 24 h using a 10 s time constant). Besides, a mobile laboratory equipped with a T100 device for sulfur dioxide (SO_2_), T200 for nitric oxides (NO_x_) (Teledyne Advanced Pollution Instrumentation, San Diego, CA, USA, precision: 0.5% of reading above 50 ppb), BAM1020 for particulate matter (MetOne Instruments, Grants Pass, OR, USA, accuracy: exceeds U.S.-Environmental Protection Agency Class III PM_2.5_ Forum on Environmental Measurements standards) and WS500 for weather condition measurements (Lufft, Fellbach, Germany, accuracy: ± 0.2 °C, ± 0.2% RH ± 0.5 hPa) was used for outdoor measurements. It was localized at the Silesian University of Technology campus and provided complex data on ambient PM_2.5_, PM_10_, SO_2_, NO_x_, NO_2,_ and NO concentrations.

The devices measured air pollutants continuously and provided 1 min averages, and these values were used to calculate the mean exposure levels during the training of each participant that was used in the statistical analysis. In addition, 3 h averages from the time prior to exercise were calculated.

For the statistical analysis, we used Statistica 13 software. The normality of the distributions of quantitative variables was checked with the Shapiro–Wilk test. Comparisons between exposure and control trials were made with a paired measurements *t*-test or Wilcoxon test, depending on the normality of the distribution of variables. For qualitative variables, we used the Fisher test. Post-exercise changes in physiological parameters were presented as relative differences—a percentage of baseline value (‘post-exercise’ – ‘baseline’ / ‘baseline’). For the correlation analysis, Spearman rank correlation was used to assess the relationship between acute post-exercise changes in physiological variables and environmental variables recorded during the exercise. Multivariable analysis was made with a logistic regression model to assess the impact of individual subjects’ characteristics and environmental conditions on the acute response in terms of FeNO. This response was the dependent variable in the model and was defined as FeNO increase during the exposure trial and decrease/no change during the control trial (outcome) or FeNO increase during both trials (no outcome). The choice of the outcome was based on the expectation that physical exercise would decrease and air pollution would increase the FeNO level. Therefore, the post-exercise increase shall be observed only during significant exposure. The explanatory independent variables included in the models were: age, BMI, physical activity level (low or high), allergy status (yes or not), and air pollutant concentration recorded during exposure trials (separate models for different pollutants). Only the pollutants that were statistically significant in simple correlation analysis were included in the regression model. The level of significance in the statistical analysis was set at *p* < 0.05. The study design was approved by the Ethics Committee of the Medical University of Silesia in Katowice (agreement no. PCN/0022/KB1/125/I/19, 3 December 2019).

## 3. Results

### 3.1. Study Group Characteristics

Participants who were eligible for the study (*n* = 76) completed at least two training trials (during the worse and better quality of ambient air) with complete FeNO and blood pressure measurements between December 2019 and March 2020. They were mostly the inhabitants of Gliwice and neighboring large cities. Of these, 31 participants (40.8%) had a high level of physical activity. The basic characteristics of the study group are presented in Table 1.

### 3.2. Environmental Conditions

During the trainings qualified as exposure trials, the concentrations of both indoor and ambient air pollutants were significantly higher than during trainings qualified as the control. The differences between exposure and control conditions are presented in Table 2.

### 3.3. Results of Health Measurements

Table 3 shows the mean differences between baseline and post-exercise measurements. The health measurements were within the normal values. Blood pressure and heart rate significantly increased and oxygen saturation decreased after exercise during both exposure and control trials. Some differences in post-exercise responses were observed between the trials in terms of FeNO. A post-exercise decrease in FeNO was statistically significant only during the control period.

The magnitude of post-exercise changes in physiological parameters is shown in Table 4. The values presented are the relative differences expressed as a percentage of the baseline value. The comparison of post-exercise changes between the exposure and control trials did not show statistically significant differences.

### 3.4. Factors Associated with the FeNO Response

Since there were no significant differences between exposure and control trials in terms of post-exercise blood pressure, heart rate, and oxygen saturation responses (Table 3), we focused on FeNO in the additional analysis.

The correlation analysis showed low but statistically significant positive correlations between post-exercise ΔFeNO during exposure trials and ambient air pollutants (NO_2_ Spearman’s ρ = 0.40, *p* < 0.001; NO_x_ ρ = 0.37, *p* < 0.001; NO ρ = 0.36, *p* = 0.001, PM_10_ ρ = 0.31, *p* = 0.007) and outdoor humidity (HUM) and atmospheric pressure (ATMP) (HUM ρ = 0.41, *p* < 0.001; ATMP ρ = 0.27, *p* = 0.01). There were no significant correlations between ΔFeNO and indoor particulate matter, nor longer exposure lags (3 h average concentrations).

In 17 participants, FeNO increased during the exposure trial, while it remained stable or decreased during the control trial. These participants were defined as the outcome group with an inflammatory response to air pollution only during significant exposure. Their trials took place when outdoor air quality was significantly worse than during the exposure trials of the rest of the study group (NO_2_ 54.2 μg/m^3^ vs. 45.3 μg/m^3^, *p* < 0.001; NO_x_ 306.3 μg/m_3_ vs. 153.8 μg/m_3_; *p* = 0.01), while indoor air quality was not significantly different (PM_10 IN_ 156.7 μg/m^3^ vs. 150.2 μg/m^3^; *p* = 0.7).

In multivariable regression analysis, FeNO increase during the exposure trials and lack of such a response during the control trials was associated only with higher physical activity level, higher concentrations of outdoor PM_10_ and NO_2_, and a higher percentage of relative outdoor air humidity recorded during the exposure trials. In comparison to participants with high activity levels, those with low activity more often had a post-exercise increase in FeNO during control trials (53.3% vs. 19.4%, *p* = 0.002). In the study group, 11 subjects had post-exercise FeNO increase during both exposure and control conditions. They were defined as the non-outcome group. Multivariable regression analysis did not show significant factors associated with such a response. However, the comparison of physical activity patterns showed that participants from the non-outcome group exercised less frequently than those from the outcome group. The environmental conditions during the study trials did not differ significantly between the groups. The results of logistic regression analysis are shown in Table 5, while Table 6 shows the basic comparison of the outcome (increase only during exposure trials) and non-outcome (increase during both trials) groups.

## 4. Discussion

Our study showed no significant differences between physiological responses to indoor training performed under different exposure conditions in young adults in terms of blood pressure, heart rate, and oxygen saturation. However, modest differences were observed for FeNO; its post-exercise decrease was statistically significant only during control trials. The post-exercise changes in FeNO during the exposure trial correlated with several outdoor, but not indoor, air pollutants.

Several other studies reported an increase in blood pressure after exercising under exposure to air pollution [13,32,33]. An explanation as to why no statistically significant differences between exposure and control trials were observed in our study in terms of blood pressure might be the time of measurement. We focused only on acute changes detectable immediately after exercise, while a blood pressure increase may require more time to develop in case of moderate aerobic exercise. It was shown that air pollution induces autonomic imbalance, reflected by decreased heart rate variability (HRV), which can be detected within minutes of exposure [34]. Such changes may prelude blood pressure increases detectable at longer time lag. Kubesch et al. observed associations between traffic-related air pollutants and SBP and DBP measured after exposure, with first measurements after 30 min of rest, but not with intraexposure measurements (during intermittent physical exercise). In contrast, Boussetta et al. reported greater SBP increases directly after exercise in a polluted area in comparison to a non-polluted location. However, in this case, the experiment consisted of high intensity anaerobic exercise [32]. 

FeNO is a marker of eosinophilic airway inflammation [20]. Physical exercise usually leads to an acute decrease in FeNO [35]. On the other hand, air pollution, especially particulate matter, induces nitrogen oxide production by pulmonary epithelial cells [36]. Several studies reported FeNO increasing after exposure to air pollution during exercise in healthy adults [16,37] and positive correlations between outdoor air pollutant levels and post-exercise changes in FeNO [38]. These effects were not observed in a study which assessed the impact of exposure to low levels of air pollution (PM_2.5_: 2–17 µg/m^3^) [25]. Our study was conducted under significantly higher exposures. This underlines the impact of air pollution levels on the pulmonary inflammatory response. In our study, higher ambient PM_10_ and NO_2_ concentrations were associated with FeNO increase during exposure trials.

It remains unclear why no associations were observed with indoor air pollutants. Most of the available studies focused on outdoor exercise and outdoor ambient air pollutants [16,25,37]. It cannot be excluded that, in terms of FeNO, outdoor exposure prior to exercise is more important than exposure during the exercise, as the inflammatory response to air pollution might require more time to develop. Chen et al. showed that in COPD patients, the strongest association between PM_2.5_ and FeNO was seen after a one day lag [36]. However, we observed no correlations between ΔFeNO and 3 h average levels of air pollutants before exercise. It should be noted that indoor air pollutants generally corresponded well with outdoor concentrations (e.g., PM_10 IN_ and PM_10 OUT_ Spearman’s ρ = 0.60, *p* < 0.001).

Physical activity level was another important factor in FeNO response to training. Active participants were more likely to have an FeNO increase only after exposure to high air pollutant levels during exposure trials. In our study, we examined different exposure scenarios, but due to the study site characteristics, we compared a high vs. low level of air pollution rather than air pollution vs. clean air. Therefore, it seems that inactive participants were more likely to present with a post-exercise FeNO increase even after exposure to low air pollutant concentrations. To the best of our knowledge, our study is the first to assess how different physical activity patterns affect acute responses to exposure to air pollution during training in young adults. However, observations in laboratory animals have provided evidence that regular aerobic exercise has a protective effect on PM-induced lung inflammation [39]. This may explain the pathophysiologic background of the observed differences.

Our study has several limitations. Firstly, the exercise trials were not standardized as they were regular physical education classes and sports training. It cannot be excluded that some training was more strenuous than others, which might influence our observations. However, in the case-crossover study, each participant serves as her/his own control. Therefore, the comparisons between exposure and control trials in general or within groups were less affected, as we may assume that different training sessions of the same section are similar. The comparisons between high and low levels of physical activity groups might be more susceptible to bias. It has been proven that with an increase in ventilation rate during the exercise, FeNO decreases [40]. As a result, different exercise intensities between groups could affect the post-exercise measurements. In addition, regular physical activity leads to a decrease in baseline FeNO [22]. Another way in which exercise intensity may affect post-exercise FeNO is the protective effect of physical activity towards airway inflammation induced by air pollution [39]. The sports training of students from a high level of physical activity group may be more strenuous than regular physical education. Therefore, the observed differences could be more pronounced in an experiment with a standardized exercise protocol. Moreover, we measured only acute post-exercise responses. Some air pollution effects might remain unobserved if they required more time to develop. Those hypotheses may require verification in a different study protocol.

The strength of our study is the study site, which is an area characterized by high air pollutant concentrations during the winter season. This enabled us to investigate effects that could be missed in lower exposure conditions. Another advantage is the relatively large study group in comparison to similar research. Also, the study protocol provided the possibility to assess the acute responses to air pollution exposure during exercise in participants with a low and high level of physical activity.

## 5. Conclusions

We found that even in young and healthy adults, some differences in physiological responses to exercise between polluted and control environments could be observed. Only exercise in control conditions was related to a significant decrease in the pulmonary inflammation marker FeNO. People with a higher level of physical activity were more likely to have an increase in FeNO only after exercise in a polluted environment and not after control exercise trials.

## Figures and Tables

**Table 1:** Anthropometric variables, physical activity patterns and allergy status of the study group (*n* = 76).

Quantitative Variable	X ± SD	Me (IQR)
Age (years)	20.9 ± 2.6	20.0 (19.0–21.0)
Height (cm)	177.4 ± 9.3	178.0 (171.3–183.3)
Body mass (kg)	74.4 ± 16.1	72.5 (62.9–84.7)
BMI	23.5 ± 4.1	23.0 (21.0–25.4)
**Qualitative variable**		***n* (%)**
Sex	Female	34 (44.7)
	Male	42 (55.3)
Place of residence	Village	6 (7.9)
	City < 100,000 inhabitants	16 (21.1)
	City ≥ 100,000 inhabitants	54 (71.0)
Physical activity level *	Low	45 (59.2)
	High	31 (40.8)
Self-perceived condition (good or very good)		48 (63.2)
Outdoor exercise during the winter season		5 (6.6)
Avoiding outdoor exercise when air quality is poor		23 (30.3)
Allergy (yes)		17 (22.4)

X—arithmetic mean, SD—standard deviation, Me—median value, IQR—interquartile range, *N*—number, * Low—university students exercising only during obligatory physical education classes, High—players training in the University Sports Association’s (Akademicki Związek Sportowy (AZS)).

**Table 2:** Distribution of environmental conditions recorded during exercise trials (*n* = 76).

Environmental Factor	Exposure Trials		Control Trials		*p* *
	Me	IQR	Me	IQR	
TEM _IN_ (°C)	21.7	20.9–22.2	22.0	20.8–22.6	0.03
HUM _IN_ (%)	34.5	25.2–51.1	38.3	33.5–54.1	0.008
PM_2.5 IN_ (μg/m^3^)	114.0	86.0–170.0	26.5	18.6–29.0	<0.001
PM_10 IN_ (μg/m^3^)	133.0	101.2–199.0	31.8	21.9–49.0	<0.001
TEM _OUT_ (°C)	1.6	0.6–5.0	4.4	2.3–12.4	<0.001
HUM _OUT_ (%)	81.6	73.9–87.6	73.0	65.6–86.4	<0.001
ATMP (hPa)	993.5	980.7–996.9	992.0	978.6–995.8	0.4
PM_10 OU_ (μg/m^3^)	106.2	81.4–149.0	34.5	30.1–47.6	<0.001
SO_2_ (μg/m^3^)	26.2	19.3–30.8	18.3	7.3–19.3	<0.001
NO_2_ (μg/m^3^)	50.6	40.0–53.2	30.5	23.7–37.9	<0.001
NO_x_ (μg/m^3^)	144.3	61.3–236.9	47.7	37.9–73.3	<0.001
NO (μg/m^3^)	61.2	12.9–120.3	12.9	8.8–18.4	<0.001

Me—median value, IQR—interquartile range, TEM _IN_ and TEM _OUT_—indoor and outdoor temperature, respectively, HUM _IN_ and HUM _OUT_—ndoor and outdoor relative air humidity, respectively, ATMP—atmospheric pressure, PM_2.5 IN_—indoor concentration of particulate matter with diameter < 2.5 μm, PM_10 IN_—indoor concentration of particulate matter with diameter < 10 μm, PM_10 OUT_—outdoor concentration of particulate matter with diameter < 10 μm, SO_2_—sulphur dioxide, NO_2_—nitrogen dioxide, NO_x_—nitrogen oxides, NO—nitrogen monoxide. * Results of Mann–Whitney U test.

**Table 3:** Mean differences between post-exercise measurements obtained in 76 participants during exposure and control trials.

Variable	Exposure Trials		Control Trials	
	Mean Difference ± SD (Post-Exercise–Baseline)	*p* *	Mean Difference ± SD (Post-Exercise–Baseline)	*p* *
FeNO (ppb)	−0.5 ± 4.3	0.3	−1.2 ± 4.1	0.03
SBP (mmHg)	5.1 ± 12.3	<0.001	6.4 ± 11.6	<0.001
DBP (mmHg)	3.0 ± 8.4	0.002	3.2 ± 8.3	0.001
HR (bpm)	24.3 ± 16.7	<0.001	24.8 ± 13.4	<0.001
SpO_2_ (%)	−1.2 ± 1.3	<0.001	−1.3 ± 1.3	<0.001

X—average, SD—standard deviation, FeNO—fractionated exhaled nitric oxide, SBP—systolic blood pressure, DBP—diastolic blood pressure, HR—heart rate, SpO_2_—oxygen saturation. *—results of paired Student’s *t*-test/Wilcoxon test.

**Table 4:** Relative differences between baseline and post-exercise measurements.

Variable	Relative Difference (%) between Post-Exercise–Baseline (X ± SD)		*p* *
	Exposure Trial	Control Trial	
ΔFeNO (ppb)	−0.19 ± 24.53	−0.14 ± 23.83	0.9
ΔSBP (mmHg)	4.47 ± 10.27	5.46 ± 9.48	0.5
ΔDBP (mmHg)	4.96 ± 12.81	4.82 ± 11.68	0.7
ΔHR (bpm)	31.46 ± 24.25	32.64 ± 20.66	0.7
ΔSpO_2_ (%)	−1.22 ± 1.22	−1.27 ± 1.28	0.8

X—average, SD—standard deviation, FeNO—fractionated exhaled nitric oxide, SBP—systolic blood pressure, DBP—diastolic blood pressure, HR—heart rate, SpO_2_—oxygen saturation. *—results of paired Student’s *t*-test/Wilcoxon test.

**Table 5:** Multivariable logistic regression analysis of increases in FeNO only during exposure trial and PM_10_, NO_2_ and air humidity.

		FeNO Increase During ExposureTrial and Decrease or No Change During Control Trial (Outcome)			
	Age * (years)	**BMI (kg/m^2^)**	**PA**	**Allergy**	**PM_10_ (μg/m^3^)**
OR **	0.851	1.077	10.248	0.655	1.013
95% CI	0.643–1.127	0.908–1.278	2.060–50.989	0.145–2.955	1.003–1.023
*P*	0.2	0.3	0.003	0.5	0.008
	**Age (years)**	**BMI (kg/m^2^)**	**PA**	**Allergy**	**NO_2_ (μg/m^3^)**
OR	0.781	1.092	7.061	0.531	1.175
95% CI	0.545–1.118	0.915–1.304	1.481–33.657	0.112–2.515	1.044–1.324
*P*	0.1	0.3	0.01	0.4	0.006
	**Age (years)**	**BMI (kg/m^2^)**	**PA**	**Allergy**	**HUM _OUT_ (%)**
OR	0.850	1.088	5.822	0.580	1.118
95% CI	0.647–1.116	0.920–1.286	1.380–24.557	0.131–2.564	1.021–1.225
*P*	0.2	0.3	0.01	0.4	0.01
	**FeNO increase during exposure trial and control trial (Non-outcome)**				
	Age * (years)	**BMI (kg/m^2^)**	**PA**	**Allergy**	**PM_10_ (μg/m^3^)**
OR **	1.025	0.892	0.500	1.376	1.002
95% CI	0.710–1.481	0.732–1.086	0.076–3.288	0.245–7.738	0.993–1.012
*p*	0.9	0.2	0.4	0.7	0.5
	**Age (years)**	**BMI (kg/m^2^)**	**PA**	**Allergy**	**NO_2_ (μg/m^3^)**
OR	1.027	0.890	0.446	1.362	0.995
95% CI	0.716–1.474	0.733–1.080	0.071–2.796	0.242–7.669	0.937–1.057
*p*	0.8	0.2	0.3	0.7	0.8
	**Age (years)**	**BMI (kg/m^2^)**	**PA**	**Allgery**	**HUM _OUT_ (%)**
OR	1.001	0.910	0.485	1.304	1.057
95% CI	0.671–1.493	0.743–1.115	0.074–3.194	0.230–7.393	0.966–1.157
*p*	0.9	0.3	0.4	0.7	0.2

* Age, BMI, PM_10_, NO_2_, HUM _OUT_—quantitative variables, PA—high/low level of physical activity—2/1, allergy—yes/no—1/2; ** OR—odds ratio, 95% CI—95% Wald confidence interval, *p*—statistical significance in Wald chi^2^ test.

**Table 6:** Comparison of the outcome and non-outcome groups based on FeNO response.

Variable		Groups Based on FeNO Response		
		Outcome (*n* = 17)	Non-Outcome (*n* = 11)	Statistical Significance
		Me	Me	*p* *
Age		21.0	20.0	0.5
HUM _OUT_ (%)	**Exposure trial**	87.6	86.0	0.4
	**Control trial**	66.3	86.2	0.2
NO_2_ (μg/m^3^)	**Exposure trial**	55.5	50.6	0.08
	**Control trial**	30.5	29.3	0.8
PM_10_ (μg/m^3^)	**Exposure trial**	149.0	143.3	0.7
	**Control trial**	30.2	36.8	0.3
		***n* (%)**	***n* (%)**	*p* **
Sex (female)		20 (58.8)	4 (36.4)	0.4
Overweight (yes)		6 (35.3)	2 (18.2)	0.4
Allergy (yes)		4 (23.5)	2 (18.2)	0.9
Physical activity level (high)		11 (64.7)	3 (27.3)	0.1
Self-perceived condition (good or very good)		14 (82.4)	8 (72.7)	0.6
Training frequency per week (4 times or more)		11 (64.7)	2 (18.2)	0.02
Average time spent on physical activity daily (2 h or more)		7 (41.2)	2 (18.2)	0.2

Me—median value, *N*—number, HUM _OUT_—outdoor air humidity, NO_2_—nitrogen dioxide, PM_10_—outdoor concentration of particulate matter with diameter < 10 μm, * Results of Mann–Whitney U test, ** Results of Fisher test.
